# Mobilizing students to effect multidisciplinary cancer care: the Tumor Board Establishment Facilitation Forum

**DOI:** 10.1016/j.lansea.2024.100441

**Published:** 2024-06-24

**Authors:** Muhammad Abdul Rehman, Urooba Jawwad, Erfa Tahir, Unaiza Naeem, Maheen Qamar, Nowal Hussain, Nimrata Kumari, Ahmed Nadeem Abbasi, Agha Muhammad Hammad Khan

**Affiliations:** a Dow Medical College, Dow University of Health Sciences, Karachi, Pakistan; b Department of Radiation Oncology, The Aga Khan University Hospital, Karachi, Pakistan; c Department of Radiation Oncology, McGill University, Montreal, Quebec, Canada

In 1995, chief medical officers, Dr. Calman and Dr. Hine established multidisciplinary tumor boards (MTB) as a standard of care in the United Kingdom.^1^ Almost 30 years later, it is a fundamental phenomenon in cancer care. However, it is not implausible to discern that not all cancer cases are discussed in MTBs. In the face of an increasing influx of new cases, the demand is extremely high for MTBs. In 2022, there were 9,800,000 new cancer cases in Asia.^2^ Yet, even nationwide MTBs only discuss cases in the hundreds.^3^^,^^4^ Additionally, MTBs are not entirely prevalent. In a Southeast Asian survey, only 24 out of 46 dedicated pediatric solid tumor institutions had an MTB.^5^ In Pakistan, due to the paucity of MTBs a philanthropic MTB had to be built.^6^

We witnessed the dearth of MTBs firsthand in our clinical rotations as medical students at the Dow Medical College in Karachi, Pakistan. MTBs were largely absent from clinical practice. Our observations were supplemented by scientific literature published by senior oncologists in the city.^6^ And so, under the mentorship of these oncologists, we brainstormed a student-led initiative titled *Tumor Board Establishment Facilitation Forum* (TEFF). The core group of students was trained through a series of leadership workshops, following which a larger team was recruited. Under TEFF, we approach doctors through emails, telephone calls, and in-person visits; handle case presentations; transfer radiology and pathology reports; host and set up meeting dates; and send MTB reminders. We evaluate MTBs using the MDT-MODe checklist and draft meeting notes.^7^ Professors from the respective ward chair the MTB. Other members are faculty members, or clinically active consultants.

On November 6, 2021, TEFF facilitated the first breast cancer MTB at the Dr. Ruth K. M. Pfau Civil Hospital in Karachi. It is one of the country’s largest public-sector tertiary care hospitals which caters to more than 1,600,000 outpatients and more than 81,000 inpatients every year.^8^ Between November 2021 and March 2024, we established three new MTBs for gynecology, head and neck, and pediatrics. Across these, TEFF facilitated the discussion of 105 cases across 50 meetings.

Our model highlights several considerations for policymakers. Firstly, scheduling meetings is difficult for MTB participants.^9^ By taking it upon ourselves to coordinate between doctors, we eliminated this burden. Secondly, we enlisted several doctors willing to contribute from all over Pakistan, thereby ensuring that a sufficient pool of doctors was always available for each meeting. Thirdly, our model is minimalistic and cheap. We utilize smartphones and laptops which are connected to the internet using available Wifi or cellular data, and meetings are held online over Google Meet. Fourthly, by focusing on our affiliated hospital we were able to hold more MTBs over a shorter span when compared to MTBs that cater to more hospitals. India’s National Cancer Grid held 54 MTBs between 2016 and 2022 and Pakistan’s pediatric neuro-oncology network held 124 MTBs between 2019 and 2023.^3^^,^^4^ We managed 50 MTBs between 2021 and 2024 at one hospital. This is largely attributable to in-person interactions and reminders which encouraged wards to discuss more cases in MTBs. Fifthly, by visiting wards in-person, we directly assessed the need for and discussed the possibility of establishing MTBs with department heads. Lastly, we maintained a pre-/post-meeting record for each case, and meeting, serving as a cancer repository prototype, which is currently absent at the national level in Pakistan.^3^

Although uncommon, we occasionally encounter poor internet connectivity, failure to show up in the MTB, and poor audio. To address these, we keep a second internet source, ensure multiple doctors are present per specialty, and facilitate the use of earphones. Establishing MTBs is a win for everyone–patients, wards, and students. Our involvement with MTBs provides a massive learning experience, especially because MTBs qualify for Continuing Medical Education (CME) credit.^9^ Their clinical relevance encouraged our medical school to incorporate one MTB meeting into the final year curriculum where medical students were spectators and could ask questions at the end. Policymakers and schools should incentivize medical students to encourage their participation in similar endeavors. In addition, MTBs should be utilized for their academic value in teaching hospitals to encourage learning and to ensure the continued existence of MTBs.

TEFF runs on a sustainable model as new medical students take over the initiative once seniors graduate. However, this also limits the applicability of our model to teaching hospitals. Nonetheless, MTBs can improve overall survival by up to 11.2 months, and no cancer patient should be withheld from this benefit.^10^ Our model is proof that calculated and coordinated interventions can improve cancer care, even with the slightest of resources.

## Contributors

Conceptualization: MAR, UJ, ET, AMHK, ANA; Data curation: MQ, NH, NK; Investigation: MAR; Project administration: MAR; Supervision: MAR, UJ; Validation: MAR, AMHK, ANA; Writing—original draft: MAR, UJ, ET, UN, NK; Writing—review & editing: MAR, UJ, ET, UN, AMHK, ANA.

## Declaration of interests

MAR, UJ, ET, UN, MQ, NH, and NK have held leadership roles at the Tumor Board Establishment Facilitation Forum (TEFF). ANA and AMHK act as mentors at TEFF.
